# Supplementary figures and images for: MicroRNAs implicated in dysregulation of gene expression following human lung transplantation
Source: Transl Respir Med. 2013 Aug 8;1:12. doi: 10.1186/2213-0802-1-12 (PMC3886917; doi:10.1186/2213-0802-1-12)

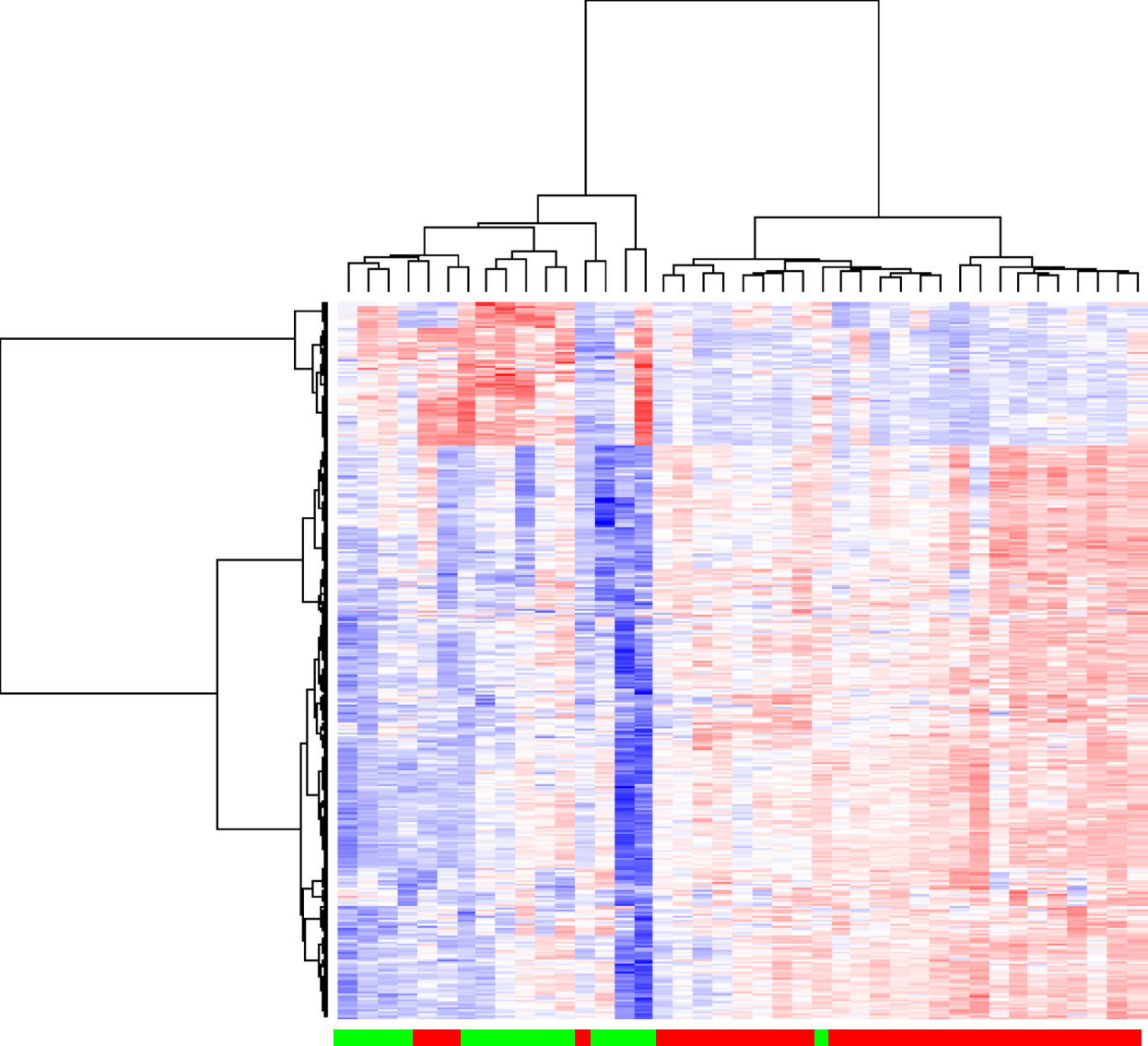

Supplement: Supplementary file 3 — Authors’ original file for figure 1 [file 40247_2013_12_MOESM3_ESM.pdf]

A

p-value=0.0046

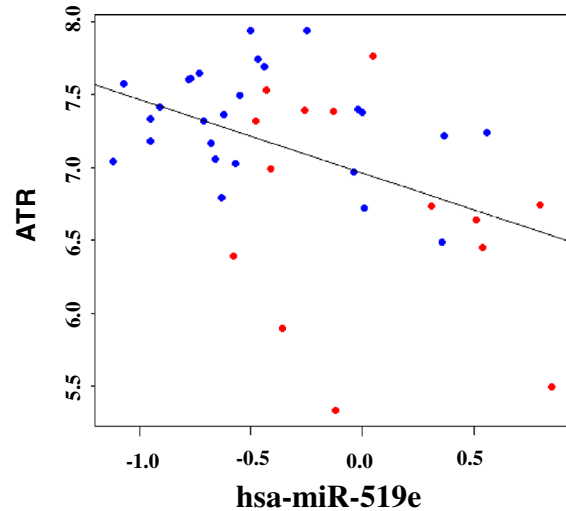

B

p-value=0.0057

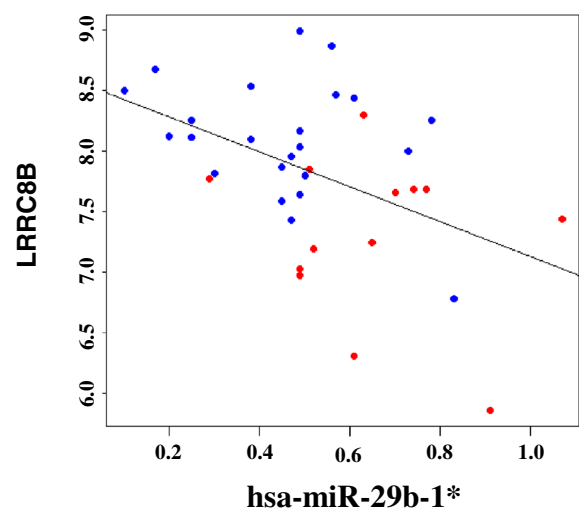

C

p-value=0.0074

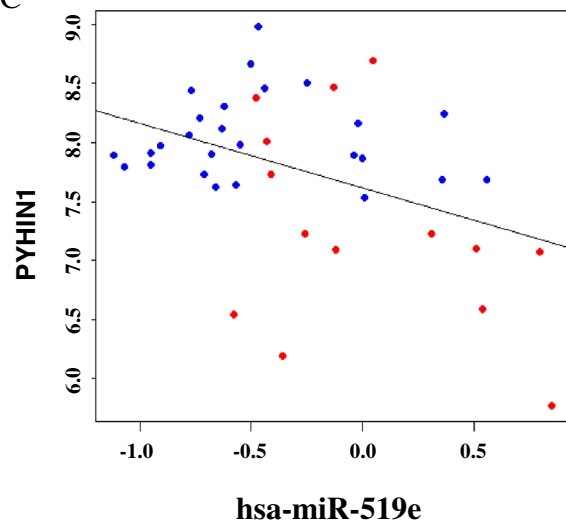

D

p-value=0.023

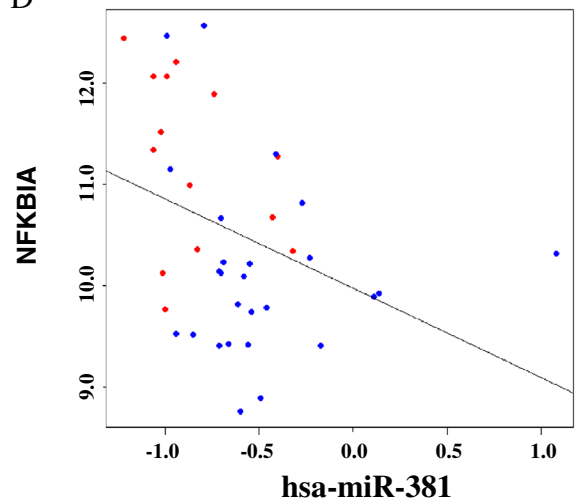

Supplement: Supplementary file 4 — Authors’ original file for figure 2 [file 40247_2013_12_MOESM4_ESM.pdf]
